# Developing human locus coeruleus organoids to study regional selective vulnerability in Alzheimer's disease

**DOI:** 10.1002/alz70855_098441

**Published:** 2025-12-23

**Authors:** Margaret M Tish, Lauren Gao, Zihui Ou, David Weinshenker, Fikri Birey

**Affiliations:** ^1^ Emory University School of Medicine, Atlanta, GA, USA

## Abstract

**Background:**

The locus coeruleus (LC), is the first brain region to develop hyperphosphorylated tau (ptau) inclusions in Alzheimer's disease (AD) and undergoes catastrophic degeneration in later disease stages. Importantly, the LC is the main noradrenergic nucleus in the brain and source of norepinephrine (NE) in the forebrain. Dysregulation of LC‐NE transmission is associated with AD symptoms, as its release in the forebrain regulates attention, arousal, stress response, and learning and memory. Moreover, the LC may transmit pathogenic tau to the forebrain via its extensive projections. However, nearly all information about the LC in AD has been derived from rodent models and postmortem or human imaging studies. It is not known how the early ptau accumulation in the human LC affects neuronal function, NE transmission, or pathology propagation. We created organoids, which are 3D human cell culture models derived from human induced pluripotent stem cells, that possess LC‐like noradrenergic neurons.

**Method:**

Organoids containing neurons resembling the LC were generated from human induced pluripotent stem cells using growth factors that induce a hindbrain fate. The cells were taken from both patients with familial AD, as well as healthy controls. These organoids were then characterized using HPLC, immunohistochemistry, RNA‐scope and RNA‐sequencing. Human cortical organoids were used for comparison.

**Result:**

LC organoids contained higher levels of NE, dopamine than cortical organoids. They also expressed neuromelanin‐like pigment and many proteins and mRNAs that are indicative of and enriched in LC‐NE neurons, including tyrosine hydroxylase, dopamine beta‐hydroxylase, the NE transporter, Phox2A, and Phox2B. Additionally, bulk RNA‐seq revealed that compared to cortical organoids, the LC organoid transcriptome more closely resembled the human LC. Bulk RNA‐seq also revealed that LC organoids derived from familial AD patients had altered gene expression reminiscent of AD pathophysiology. Finally, AD LC organoids showed increased 4R:3R tau ratio compared to control LC organoids.

**Conclusion:**

Human LC organoids contain LC‐like noradrenergic neurons that show pathogenic alterations when generated from familial AD mutation carriers. Studying the structure and function of LC organoids derived from both healthy controls and AD patients may provide new insights into the role of the LC and biology of selective vulnerability in AD.

